# Efficacy of Chlorobenzene as a New Fumigant for Control of Confused Flour Beetle (Coleoptera: Tenebrionidae) and Rice Weevil (Coleoptera: Curculionidae)

**DOI:** 10.3390/insects16020183

**Published:** 2025-02-08

**Authors:** Yong-Biao Liu

**Affiliations:** Sam Farr United States Crop Improvement and Protection Research Center, Agricultural Research Service, United States Department of Agriculture, Salinas, CA 93905, USA; yongbiao.liu@usda.gov; Tel.: +1-(831)-755-2825

**Keywords:** chlorobenzene, fumigation, stored product insects, postharvest pest control, *Tribolium confusum*, *Sitophilus oryzae*

## Abstract

Chlorobenzene is an industrial chemical with relatively high vapor pressure and has been used in the past to produce pesticide DDT (Dichlorodiphenyltrichloroethane). In this study, chlorobenzene was demonstrated to be an effective fumigant against two stored product insects: the confused flour beetle (*Tribolium confusum*) and rice weevil (*Sitophilus oryzae*). In small-scale fumigations in 1.9 L glass jars, the complete control of adults of both the confused flour beetle and rice weevil was achieved in 24 h at a dose of 150 μL/L at 21 °C. LC_95_ values of chlorobenzene vapor concentration for adults of the confused flour beetle and rice weevil were estimated to be 1121 and 1114 ppm, respectively. In large-scale fumigations in a 60 L chamber, all life stages of the confused flour beetle and rice weevil in 20 kg of corn were fumigated for 24 h with 30 mL (500 μL/L) chlorobenzene at 21 °C. The complete control of adults and immature stages of the confused flour beetle was achieved. For the rice weevil, adults had 100% mortality, and immature life stages had 97.8% mortality. These results demonstrated that chlorobenzene is effective as a fumigant against stored product insects, and it is technically feasible to conduct large-scale fumigations for postharvest pest control on stored products.

## Simple Summary

There is a critical lack of safe and effective alternative treatments to replace methyl bromide fumigation for postharvest pest control on fresh and stored products. Current alternative fumigants, including phosphine and sulfuryl fluoride, have serious shortcomings in meeting the needs of postharvest pest control, and new fumigants are urgently needed. In this study, chlorobenzene, an industrial solvent chemical, was discovered to be an effective fumigant against two major stored product insects: confused flour beetle and rice weevil. In fumigation trials of 20 kg of corn in a 60 L fumigation chamber, the complete control of all life stages of the confused flour beetle was achieved. For rice weevil, the complete control of adults and 97.8% mortality of immature life stages were achieved in 24 h fumigations. These results indicated the high efficacy of chlorobenzene against stored product insects and the promising feasibility of being used on stored products for postharvest pest control. Because chlorobenzene has low toxicity to mammals, high volatility, and is commercially available at a low cost, it has good potential to be used as a safe, effective, and economical alternative fumigant for postharvest pest control and, therefore, warrants continued research and development efforts in the future.

## 1. Introduction

Postharvest pest control is critical for stored products. In the past, methyl bromide fumigation was the primary method to control postharvest pests on stored products. Since methyl bromide production has been phased out globally, alternative fumigants are needed. Phosphine and sulfuryl fluoride are two major alternative fumigants, but they cannot meet the needs for postharvest pest control due to their respective shortcomings. Phosphine fumigation has long treatment times (>10 days for some insects) and is not effective against some pests due to the emergence of phosphine-resistant pest populations and a natural tolerance to phosphine fumigation [1]. Sulfuryl fluoride is mainly for structure fumigation [2], and sulfuryl fluoride fumigation is not effective against insect eggs [3]. Newer alternative fumigants, including ethyl formate, have shown promise to control postharvest pests on some fresh commodities but also pose phytotoxicity to many other fresh products [4,5]. Recent studies also show that nitric oxide [6,7] and sulfur dioxide (SO_2_) [8] have high efficacy against postharvest pests on fresh and stored products. It is not clear if nitric oxide will be used commercially due to its small niche market of chamber fumigation and the high cost of registration. SO_2_ fumigation may cause injuries to some fresh products [9]. More safe and effective alternative fumigants are urgently needed to meet the needs of postharvest pest control.

In recent years, there have been intensive research efforts on plant essential oils to discover and develop more environmentally friendly pesticides, including fumigants [10,11,12,13,14,15,16,17,18,19]. Compared with most plant essential oils, industrial organic volatile compounds are much smaller molecules and have higher vapor pressures, making them suitable to function as fumigants. Several organic volatile oil compounds were reported to be effective fumigants against insects. They include methyl benzoate, anisole, and cyclohexanone [20,21,22].

Among the three organic volatile compounds, methyl benzoate has the lowest vapor pressure of 0.38 mmHg at 25 °C and is better functioning as a contact pesticide rather than a fumigant [20]. Anisole and cyclohexanone have much higher vapor pressures of 3.54 and 5.2 mmHg at 25 °C, respectively, and are more effective than methyl benzoate as fumigants [21,22]. Due to their very low vapor pressures compared with traditional fumigants, there is a lack of research on organic volatile compounds as fumigants. Also, due to their volatility, there is also a lack of research on organic volatile compounds as pesticides. For example, cyclohexanone has been used extensively as a solvent for pesticides but has not been reported as a pesticide itself until recently [22]. Therefore, organic volatile compounds present opportunities to discover new fumigants for pest control.

Chlorobenzene is also known as monochlorobenzene, benzene chloride, chlorobenzol, and phenyl chloride. It is a colorless organic oil compound with the formula C_6_H_5_CI; the molecular weight is 112.56. It is practically insoluble in water (water solubility: 466.3 mg/L). It is flammable with a flash point of 27–29.4 °C (EPA category 3). It has a vapor pressure of 11.7 mmHg at 20 °C. Its vapor density is 3.9 relative to air (air = 1). Chlorobenzene is not carcinogenic and has a very low oral and dermal toxicity (EPA category 4), with an OSHA exposure limit of 75 ppm for an 8 h workday and 40 h workweek. Chlorobenzene is used as an intermediate in the manufacture of rubber products and as a solvent in adhesives, paints, polishers, and others. In the past, chlorobenzene was used to produce the pesticide DDT (Dichlorodiphenyltrichloroethane). No publications have been found that show that chlorobenzene was used in pest control [23,24,25].

In this study, chlorobenzene was evaluated as an alternative fumigant for effects on mortality of two stored product insects: confused flour beetle, *Tribolium confusum* Jacquelin du Val (Coleoptera: Tenebrionidae) and rice weevil, *Sitophilus oryzae* (L.) (Coleoptera: Curculionidae). The confused flour beetle and rice weevil are two major stored product insects on stored grains.

## 2. Materials and Methods

### 2.1. Chemical and Insects

Chlorobenzene (99.8% purity) was obtained from MilliporeSigma (Burlington, MA, USA). Confused flour beetles and rice weevils were maintained on a commercial wheat flour diet (Josh’s Frogs LLC, Owosso, MI, USA) and pearled barley, respectively, in glass jars at room temperature on laboratory benches. New colonies were established as needed by transferring confused flour beetle and rice weevil adults from existing colonies to jars with wheat flour diet and pearled barley, respectively.

### 2.2. Fumigation Against Adults of Confused Flour Beetle and Rice Weevil

Confused flour beetle and rice weevil adults were fumigated with chlorobenzene at doses of 25, 50, 100, and 150 μL/L to determine the effects of different doses and vapor concentrations on insect mortality. Adults of the two species were collected separately in small 49 mL plastic vials (3 cm diam. by 7 cm high) from their prospective colonies. Each vial had about 50 adult insects without food. One vial with confused flour beetle adults and one vial with rice weevil adults were placed in a 1.9 L jar for a fumigation treatment. The lid of each fumigation jar had two ports equipped with stopcocks. One port underneath the lid was connected to a tube that extended and opened to the middle of the jar. A filter paper disk (5.5 cm diameter) was clipped to the other port without a tube underneath the lid. Chlorobenzene liquid was injected, passing through a stopcock using a syringe with a long needle, through the port not connected to the tube to deposit chlorobenzene liquid onto the filter paper disk to initiate fumigation.

**Figure 1:**
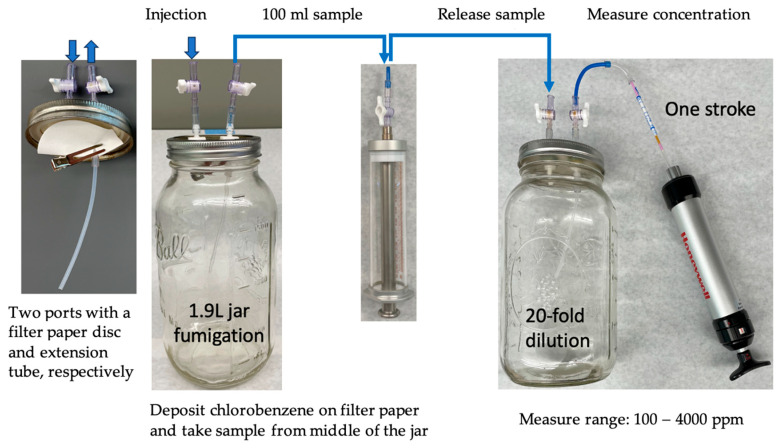
Procedures for measuring chlorobenzene vapor concentrations using chlorobenzene detector tubes.

After injection, jars were placed in a fume hood at 21 °C for 24 h to complete a fumigation treatment. Chlorobenzene vapor concentrations were measured using chlorobenzene detector tubes with a 2–500 ppm range (Gastec Corp., Kanagawa, Japan) at 2 and 6 h after the start and at the end of fumigation. A 100 mL air sample was taken through the port with a tube from the middle of each jar using an airtight syringe; the air sample was then injected into a 1.9L jar to have the sample diluted 20-fold. Then, 100 mL of diluted air sample was drawn through a detector tube using a hand pump (Model LP-1200, Honeywell Analytics Inc., Lincolnshire, IL, USA) to measure chlorobenzene vapor concentrations (Figure 1). Unfumigated adults held at 21 °C were used as controls. After fumigation, the insects were kept at room temperature overnight before being scored for mortality. Each treatment was replicated 3–5 times. Totals of 1136 confused flour beetle adults and 1147 rice weevil adults were used.

### 2.3. Large-Scale Fumigations for Controlling All Life Stages of Confused Flour Beetle and Rice Weevil on Corn

**Figure 2:**
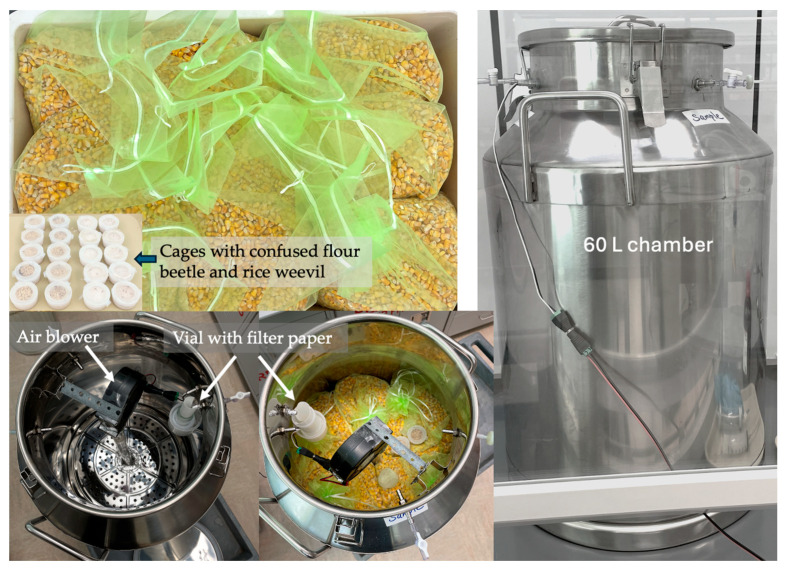
Setup of chlorobenzene fumigation of 20 kg corn with all life stages of confused flour beetle and rice weevil in a 60 L fumigation chamber.

Chlorobenzene fumigations of corn with confused flour beetle and rice weevil were conducted in a 60 L fumigation chamber to demonstrate the efficacy and technical feasibility of large-scale fumigations (Figure 2). The chamber was modified from a 60 L diary pot (38 cm diameter × 62 cm high, 25 cm opening, Eapmic Brand, Amazon.com). A 38 cm diameter round stainless-steel steamer rack was positioned about 7 cm above the bottom to form a raised platform. A 2 cm diameter × 40 cm long plastic tube was positioned vertically with one end fixed to a hole at the center of the platform. A 12Vdc air blower (SparkFun Blower—Squirrel Cage, Sparkfun Electronics, Niwot, CO, USA) was connected to the top end of the tube. Three ports were added on the neck of the diary pot for electricity supply, liquid injection, and gas sampling. A 45 mL Nalgene bottle (Thermo Fisher Scientific, Waltham, MA, USA) with a rolled filter paper disc was positioned below one of the ports to serve as a reservoir for chlorobenzene injected. The bottle was also positioned close to the air inlet side of the air blower. 

A total of 20 kg of corn was placed in 9 nylon mesh bags (30 cm × 40 cm). Confused flour beetle adults and other life stages with a flour diet and rice weevil adults and other life stages with pearled barley were sealed in 63.6 mL (4.5 cm diam. × 4 cm high) plastic cylinder cages with mesh screen tops and bottoms. A total of 15 cages were set up for each species for each treatment. One cage of confused flour beetles rearing medium with all life stages and one cage with all life stages of rice weevils was placed in each mesh bag with corn. The bags were packed in the chamber in two layers. Two cages with confused flour beetles and rice weevils, respectively, were placed on top of the corn bags. Ten cages for each species were set up to be fumigated. Five cages for each species were used as controls. The chamber was then sealed and placed in a fume hood. An aliquot of 30 mL of chlorobenzene was injected into the bottle with filter paper in the chamber through the port directly above the bottle, and the air blower was turned on to start a fumigation treatment. The fumigation treatment lasted for 24 h at 21 °C.

In each fumigation, chlorobenzene vapor concentrations were measured at 2, 4, 6, 9, 21, and 24 h using chlorobenzene detector tubes. For each measurement, a 40 mL air sample from the headspace of the chamber was taken and diluted 50-fold in a 1.9 L glass jar. Then, chlorobenzene detectors were used to measure chlorobenzene concentrations. At the end of each fumigation, the lid of the fumigation chamber was removed, and the air blower was kept running for at least 2 h to ventilate the chamber. The corn bags were then removed and cages with insects were recovered. Insects, together with diet from each cage, were placed in a plastic vial and sealed with a screened lid. All adults were removed from each vial, and the mortality of adults was scored the next day. All vials with a diet containing other life stages were incubated at 25 °C in an environmental chamber to allow surviving immature individuals to develop and emerge as adults. The vials were checked weekly to count and remove emerged adults to assess mortality of immature stages until there was no adult emergence for two consecutive weeks. The large-scale fumigation test was replicated three times.

### 2.4. Data Analysis

Mortality data for adults were transformed by arcsine$\sqrt{x}$ prior to statistical analysis. A one-way ANOVA and Tukey’s HSD multiple range test were used to compare adult mortalities among different doses of chlorobenzene using the Fit model of JMP Statistical Discovery software v16 [26]. Average vapor concentrations for all doses were calculated based on measurements with chlorobenzene detector tubes. Regression analyses were carried out between angular transformed (arcsine$\sqrt{x}$) mortality values and logarithms of chlorobenzene vapor concentrations (ppm) for confused flour beetle and rice weevil adults using the Fit y by x analysis of JMP statistical software v16 [26]. LC_95_ (Lethal Concentration for 95% mortality) values of chlorobenzene vapor concentrations for adults of the two insects were calculated using the regression lines. For immature life stages in the large-scale fumigations, relative mortality for the treatment was calculated based on adult emergence from controls, assuming each cage had the same number of insects for each species for comparison between the treatment and the control.

## 3. Results

**Figure 3:**
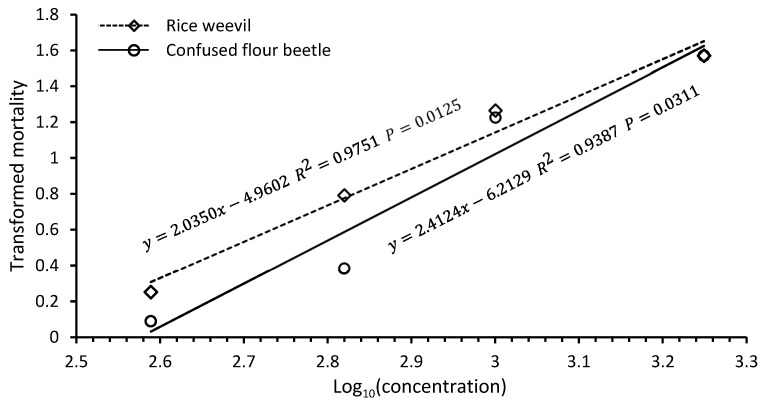
Relationship between mortality values transformed by arcsine$\sqrt{x}$ and logarithms of chlorobenzene vapor concentrations (ppm) for confused flour beetle and rice weevil adults in 24 h fumigations in 1.9 L jars at 21 °C.

Chlorobenzene fumigation was effective against both confused flour beetles and rice weevils. However, there were also considerable differences among species and life stages in susceptibility to chlorobenzene fumigation. In tests against confused flour beetle and rice weevil adults in jars, the complete control of both species was achieved in 24 h fumigations with 150 μL/L of chlorobenzene. There were significant differences in mortality among different doses for both species (Table 1). At 50 and 100 μL/L doses, confused flour beetle adults had 13.9 and 88.7% mortalities, respectively, and rice weevil adults had 50.8 and 91.1% mortalities, respectively, suggesting that rice weevil adults were more susceptible to chlorobenzene fumigation than confused flour beetle adults (Table 1). The average chlorobenzene vapor concentrations were 388, 660, 1000, and 1775 ppm for the 25, 50, 100, and 150 μL/L doses, respectively. Mortality values transformed by arcsine$\sqrt{x}$ were highly correlated with logarithms of chlorobenzene vapor concentrations for both confused flour beetle adults (y=2.4124x−6.2129, *R*^2^ = 0.9387, *p* = 0.0311) and rice weevil adults (y=2.0350x−4.9602, *R*^2^ = 0.9751, *p* = 0.0125) (Figure 3). The *R*^2^ values are the proportions of mortality variations explained by the equations. The LC_95_ values of chlorobenzene vapor concentrations were estimated to be 1358 and 1254 ppm for confused flour beetle and rice weevil adults, respectively, based on the regression equations.

Effective control of both confused flour beetles and rice weevils was also demonstrated in the large-scale fumigations of corn. The complete control of adults and immature life stages was achieved against the confused flour beetle. For the rice weevil, the complete control of adults was achieved. For the immature life stages of the rice weevil, a total of 1645 adults emerged from 15 control vials, and a total of 73 adults emerged from 30 treatment vials. Assuming equal numbers of vials for the control and the treatment, the relative mortality of the immature life stages measured by adults who emerged was calculated to be 97.8% (Table 2). These results suggest that chlorobenzene fumigation had higher efficacy against rice weevil adults than in immature life stages.

**Figure 4:**
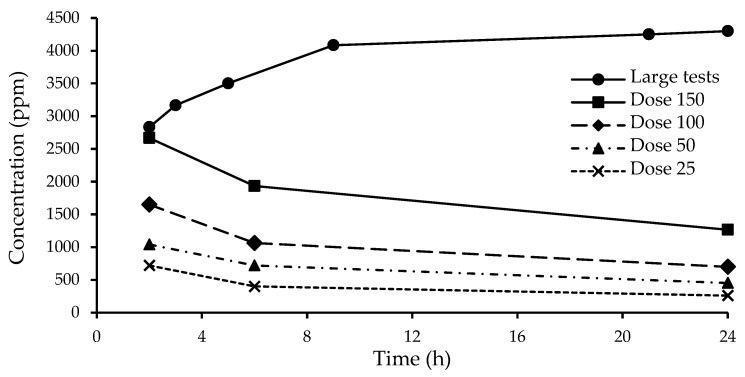
Chlorobenzene vapor concentrations measured with detector tubes at different times in large-scale fumigations with 30 mL of chlorobenzene in a 60 L chamber and in small-scale fumigations in 1.9 L jars with 25, 50, 100, and 150 μL/L doses of chlorobenzene.

In small-scale fumigations in jars against adults, chlorobenzene vapor concentrations had the highest values at 2 h after injection and then showed steady declines and reached <50% of the initial values at the end of 24 h fumigations (Figure 4). The average of chlorobenzene vapor concentrations in the large-scale tests, however, increased over time and reached the highest level of about 4300 ppm at the end of 24 h fumigations (Figure 4). All 30 mL of chlorobenzene in each of the three large-scale fumigation tests was vaporized by the end of the fumigation.

## 4. Discussion

Chlorobenzene fumigation was demonstrated to be effective against confused flour beetles and rice weevils. The effective control of confused flour beetles and rice weevils in 24 h fumigations suggested that chlorobenzene is a promising alternative fumigant against stored product insects. The effective control of the two insects in the large-scale fumigations of corn demonstrated technical feasibility and showed that chlorobenzene fumigation has good potential to be developed and used on a commercial scale to control postharvest pests on stored products. This study was also the first case in which chlorobenzene was demonstrated to be insecticidal. Previously, larvae of the midge *Chironomus riparius* were reported to exhibit behavioral responses to exposure to chlorobenzene in aquatic medium [27].

In small-scale fumigations in jars, chlorobenzene vapor concentrations were highest in the initial measurements 2 h after injections and then declined over time and reached the lowest levels at the end of 24 h fumigations (Figure 4). These results suggested that chlorobenzene vaporized rapidly after injection and vapor then likely gradually condensed on surfaces of fumigation jars and materials in the fumigation jars. Chlorobenzene vapor is not stable in air due to photodegradation with a half-life as short as 20–40 h [23,28]. However, fumigations were conducted under low light conditions in a fume hood, and photodegradation of chlorobenzene vapor is not expected to contribute to the decline of chlorobenzene vapor concentration during fumigation. In the large-scale fumigations, however, chlorobenzene vapor concentrations increased over time (Figure 4). This was likely due to the small surface area of the filter paper rolled in the chlorobenzene vial that restricted the vaporization rate of chlorobenzene liquid from the vial. The strong correlations between angular transformed mortality data and logarithms of chlorobenzene vapor concentrations indicate chlorobenzene detector tubes can be used to monitor and determine effective vapor concentrations of chlorobenzene in fumigation treatments. The LC_95_ values of 1358 and 1254 ppm for confused flour beetle and rice weevil adults, respectively, suggest that chlorobenzene as a fumigant is highly effective against insects.

In large-scale fumigations, chlorobenzene fumigation was more effective against rice weevil adults than immature life stages. It is not clear whether immature life stages are more tolerant to chlorobenzene than adults. Rice weevil adults live in spaces outside grains. Immature life stages, including egg and larva, are partially burrowed inside grains. This may reduce exposure to chlorobenzene vapor during fumigation and lead to lower mortality than adults. The complete control of adults and immature life stages of confused flour beetles, the complete control of rice weevil adults, and >97% mortality of rice weevil immature life stages in large-scale fumigation indicate that chlorobenzene vapor can penetrate well through stored corn to control stored product insects with active air circulation as demonstrated in this study.

In this study, only a 24 h treatment time was used to demonstrate the high efficacy of chlorobenzene as a fumigant for postharvest pest control. In practice, treatment time can be extended to control more tolerant pests. The large-scale tests were also intended to demonstrate the technical feasibility of practical applications of chlorobenzene fumigation against stored product insects on stored products. For practical applications, chlorobenzene dose and treatment time can be adjusted depending on pest tolerance and environmental conditions. For example, the current large-scale fumigation tests can be extended significantly to ensure successful control of target pests.

Chlorobenzene has a flash point of 27 °C. Its vapor has an explosion range between 1.8% and 9.6%. This means that fumigation should be conducted at a temperature below 27 °C to be safe when an excessive amount of chlorobenzene is used. Otherwise, the amount of chlorobenzene released must be smaller than the amount that will result in 1.8% vapor in the air. A dose of about 80 μL/L is expected to generate 1.8% vapor when fully vaporized, assuming no sorption on substrates. Because of its low vapor pressure compared with almost all other fumigants, active air circulation is necessary to assist the vaporization of chlorobenzene liquid and to help the dispersion and penetration of chlorobenzene vapor through fumigated products. The successful control of confused flour beetles and rice weevils in the large-scale fumigation of corn suggests that commercial-scale chlorobenzene fumigation is technically feasible and has good potential to be developed for practical applications.

In this study, the vapor concentrations of chlorobenzene in the large-scale fumigation that effectively controlled the two stored product insects were less than 5000 ppm (25 mg/L). However, the applied dose of chlorobenzene liquid was 500 μL/L (30 mL in a 60 L chamber) and would result in about 11% vapor concentration if completely vaporized without sorption. The vast difference between the measured 4300 ppm vapor concentration and the 11% expected vapor concentration indicated that >95% of chlorobenzene was adsorbed on the 20 kg of corn in the chamber. The adsorption rate was calculated to be 1.58 g/kg. A period of post-treatment aeration will be needed for desorption.

In comparison with phosphine, which is the most widely used alternative to methyl bromide for postharvest pest control on stored products, chlorobenzene fumigation has higher efficacy due to its shorter treatment time of 24 h in this study than several days of treatment time against stored product insects for phosphine fumigations [1]. A dose of phosphine as high as 12.7 mg/L (8368 ppm) was needed to control phosphine-resistant lesser grain borer (*Rhyzopertha dominica*) in 24 h fumigations [29]. As the loss of efficacy of phosphine fumigation against phosphine resistance in stored product insect populations becomes a serious problem, chlorobenzene fumigation can be a potential treatment for phosphine-resistant insect populations.

It is not clear what the mode of action of chlorobenzene is against insects. However, chlorobenzene was used to synthesize DDT and is the major structure of the DDT molecule. Each DDT molecule has two chlorobenzene molecules. When a chlorine atom on either chlorobenzene in a DDT molecule is replaced with other molecular structures, the toxicity of the modified DDT molecule (analog) is changed [30,31], suggesting chlorobenzene structure is vital for toxicity of DDT and its mode of action might be similar to DDT. DDT acts primarily on the peripheral nervous system. The mechanism of DDT toxicity is to prevent the deactivation or closing of sodium gates of the axon after activation and membrane depolarization. This leads to a lingering leakage of Na+ ions through the nerve membrane, creating a destabilizing negative afterpotential. The hyperexcitability of the nerve results in repetitive discharges in the neuron at or after a single stimulus [32,33].

Pesticides such as DDT with chlorinated hydrocarbon structures have very low water solubility and very high lipid solubility. They are also very resistant to degradation and persistent for a long time in the environment [32,33]. Chlorobenzene in air, however, has a half-life of about 9 days and 20–40 h, according to different authors [28]. Therefore, chlorobenzene fumigation is unlikely to leave significant residues in fumigated products.

Chlorobenzene is widely used in industry and has high permittable exposure levels. The Occupational Safety and Health Administration (OSHA) has established a legally enforceable maximum limit of 75 ppm of chlorobenzene in workplace air for an 8 h/day, 40-h work week [23]. Therefore, chlorobenzene fumigation is also relatively safer to operators compared with fumigations using most other fumigants.

Due to the global phaseout of methyl bromide production, as it depletes atmospheric ozone and shortcomings of current major alternative fumigants phosphine and sulfuryl fluoride [1,3], chlorobenzene has the potential to become an environmentally friendly, effective, and economical alternative fumigant for postharvest pest control. Chlorobenzene fumigation acted fast against insects with an effective treatment time of 24 h against confused flour beetles and rice weevils. Ethyl formate fumigation is phytotoxic to most fresh products [4,5] and has high water solubility and, therefore, has limited potential for postharvest pest control. Chlorobenzene has not been studied for phytotoxicity to fresh products. More studies are needed to determine the efficacy of chlorobenzene fumigation against different pest species as well as against different life stages and its potential impact on the postharvest quality of fresh products.

The advantages of chlorobenzene as a fumigant for pest control include high efficacy against pests, relatively low mammalian toxicity, and low cost. As an EPA category 4 chemical, chlorobenzene is much safer than most pesticides. Chlorobenzene is available from multiple vendors, and the wholesale price was listed at about 1–1.5 USD/kg [34]. All available information suggests that chlorobenzene has good potential to be an effective, safer, and economical fumigant for pest control and can have a meaningful impact on pest control and contribution to food safety.

## 5. Conclusions

Chlorobenzene is a newly discovered fumigant for pest control. In this study, 24 h fumigations with chlorobenzene were demonstrated to be effective against confused flour beetle and rice weevil. Because chlorobenzene is an industrial chemical with very low toxicity to mammals and low costs and degrades rapidly in the environment, it has good potential to be used as an environmentally friendly fumigant for postharvest pest control.

## Figures and Tables

**Table 1:** Effects of 24 h chlorobenzene fumigations at different doses on mortality of confused flour beetle and rice weevil adults at 21 °C.

Chlorobenzene (µL/L)	Confused Flour Beetle Adults		Rice Weevil Adults	
	Total	Mortality (%) Mean ± SE	Total	Mortality (%) Mean ± SE
Control	261	0 c	259	1.1 ± 0.7 e
25	253	0.8 ± 0.8 c	257	6.2 ± 1.2 d
50	256	13.9 ± 5.0 b	260	50.8 ± 4.8 c
100	211	88.7 ± 6.0 a	212	91.1 ± 3.0 b
150	155	100 a	159	100 a
		DF = 4, 17		DF = 4, 17
ANOVA		F = 171.2590		F = 239.6679
		*p* < 0.0001		*p* < 0.0001

Mortality values for each species were transformed by arcsine$\sqrt{x}$ prior to one-way analysis of variance (ANOVA). For each species, mortality values followed by the same letter were not significantly different based on Tukey’s multiple comparison test (*p* > 0.05) [26].

**Table 2:** Mortality of adults and immature life stages of confused flour beetle and rice weevil in 24 h large-scale fumigations of 20 kg of corn with chlorobenzene at a dose of 500 μL/L in a 60 L chamber at 21 °C.

Species	Treatment	Adults		Immature Stages	
		Total	Mortality (%) Mean ± SE	Survived to Adults	Relative Mortality (%)
Confused flour beetle	Control	301	14.8 ± 2.5	140	0
	Chlorobenzene	931	100	0	100
Rice weevil	Control	910	6.2 ± 1.6	1645	0
	Chlorobenzene	1294	100	73	97.8

## Data Availability

Data are available upon request.
